# TBK1 Limits mTORC1 by Promoting Phosphorylation of Raptor Ser877

**DOI:** 10.1038/s41598-019-49707-8

**Published:** 2019-09-17

**Authors:** Ricardo J. Antonia, Johnny Castillo, Laura E. Herring, D. Stephen Serafin, Pengda Liu, Lee M. Graves, Albert S. Baldwin, Robert S. Hagan

**Affiliations:** 1 Lineberger Comprehensive Cancer Center, University of North Carolina at Chapel Hill, Chapel Hill, NC USA; 2 UNC Proteomics Core Facility, Department of Pharmacology, University of North Carolina at Chapel Hill, Chapel Hill, USA; 3 Department of Cell Biology and Physiology, University of North Carolina at Chapel Hill, Chapel Hill, USA; 4 Department of Biochemistry and Biophysics, University of North Carolina at Chapel Hill, Chapel Hill, USA; 5 Division of Pulmonary Diseases and Critical Care Medicine, Department of Medicine, University of North Carolina, Chapel Hill, NC 27599 USA; 6 Marsico Lung Institute, University of North Carolina at Chapel Hill, Chapel Hill, NC 27599 USA; 7 Present Address: The Helen Diller Family Comprehensive Cancer Center, The University of California San Francisco, San Francisco, California USA

**Keywords:** Kinases, Cell signalling, Innate immunity

## Abstract

While best known for its role in the innate immune system, the TANK-binding kinase 1 (TBK1) is now known to play a role in modulating cellular growth and autophagy. One of the major ways that TBK1 accomplishes this task is by modulating the mechanistic Target of Rapamycin (mTOR), a master regulator that when activated promotes cell growth and inhibits autophagy. However, whether TBK1 promotes or inhibits mTOR activity is highly cell type and context dependent. To further understand the mechanism whereby TBK1 regulates mTOR, we tested the hypothesis that TBK1 phosphorylates a key component of the mTOR complex 1 (mTORC1), Raptor. Using kinase assays coupled with mass spectrometry, we mapped the position of the TBK1 dependent phosphorylation sites on Raptor *in vitro*. Among the sites identified *in vitro*, we found that TBK1 promotes Raptor Ser877 phosphorylation in cells both basally and in response to pathogen-associated molecules known to induce TBK1 activity. The levels of Raptor Ser877 phosphorylation were inversely correlated with the levels of mTOR activity. Expression of a mutant Raptor that could not be phosphorylated at Ser877 led to an increase in mTORC1 activity. We conclude that TBK1 limits mTORC1 activity by promoting Raptor Ser877 phosphorylation.

## Introduction

TANK-binding kinase 1 (TBK1) is an IKK-related kinase that is known for its role in the innate immune system^1^. In response to pathogens and pathogen-associated molecules, TBK1 is recruited to the Interferon Regulatory Factor 3 (IRF3) transcription factor by adaptor molecules including TANK, STING, and TRIF. TBK1 then phosphorylates IRF3 at multiple serines including Ser396, which promotes its DNA binding and transcription activation potential. IRF3, in turn, promotes transcription of type I interferons and other host proteins to promote pathogen clearance^1^. Beyond its role in innate immunity, TBK1 is known to be necessary for the pathology of diseases including cancer^2,3^ and metabolic syndromes such as obesity and diabetes^4^.

The importance of TBK1 in these diseases may be due in part to its ability regulate the mechanistic Target of Rapamycin (mTOR)^5,6^, a kinase known as a master regulator of cell growth^7,8^. When mTOR is active, it promotes cell growth primarily by stimulating protein synthesis and inhibiting autophagy, but also regulates a myriad of cell processes related to cell growth and metabolism^7^. mTOR is found in two distinct complexes, mTORC1 and mTORC2. In addition to mTOR, mTORC1 contains regulatory proteins Raptor, MLST8, PRAS40, and DEPTOR^7^. Raptor (regulatory associated protein of mTOR) promotes mTORC1 kinase activity and substrate specificity by binding to TOS-motif containing proteins and directing them towards the active site of the mTOR kinase domain for phosphorylation^9^. The canonical substrates for mTORC1 include several proteins that regulate protein translation (e.g. pS70 S6K and 4EBP-1) and autophagy (e.g. Ulk1). mTORC2 on the other hand primarily phosphorylates Akt, PKC, and SGK1^10^.

While mTORC1 signaling is most well-characterized in response to changes in growth factors and nutrients, it is now appreciated that many different signaling pathways, including those that activate TBK1, converge on mTOR. However, the mechanism and consequences of TBK1-mediated mTOR regulation remain incompletely understood and are likely to be highly context-dependent. Several publications indicate that TBK1 may repress mTOR activity, while others indicate that TBK1 may activate mTOR. For example, overexpression of wild-type but not kinase-dead TBK1 was sufficient to block mTORC1 phosphorylation of p70S6K in prostate cancer cells and TBK1-mediated suppression of mTOR was linked to prostate cancer dormancy^6^. In a model of chronic immune activation induced by knockout of the Three-prime repair exonuclease 1 (Trex1), TBK1 was linked to suppression of mTORC1 activity^11^. TBK1 is also known to promote autophagy, a process that is strongly opposed by mTORC1^12^. In contrast, several publications indicated that TBK1 promotes activation of the upstream mTORC1 activator, Akt^13^ and one recent report demonstrated that TBK1 increases mTORC1 activity through direct phosphorylation of mTOR itself^5^.

When a large panel of lung cancer cell lines was analyzed for their sensitivity to TBK1 inhibition, the authors observed a wide range of sensitivity of the cell lines to TBK1 inhibitors, with some cell lines being very resistant to TBK1 inhibition and others being highly sensitive to TBK1 inhibition^3^. Interestingly, in cell lines that were sensitive to TBK1 inhibition, TBK1 inhibitors decreased markers of mTOR activity, whereas the opposite occurred in TBK1 inhibitor resistant cell lines. This observation indicates that TBK1 activity has the opposite effect on mTOR activity in different cell lines. This contradiction, where some studies suggest that TBK1 activates mTORC1 while and others suggest that TBK1 inhibits mTORC1, indicates that the role of TBK1 in mTORC1 regulation is complex and context-dependent.

To further understand the relationship between TBK1 and mTOR signaling we tested whether TBK1 phosphorylates a critical component of the mTORC1 complex, Raptor. Raptor is regulated by phosphorylation on multiple sites by a diverse set of signaling cascades and kinases, and this allows for fine-tuning of mTORC1 signaling^14,15^. Using a proteomics approach, we found that TBK1 regulates Raptor Ser877 phosphorylation, both in cell-free kinase assays and in intact cells. TBK1 regulated Raptor Ser877 both basally in mouse embryonic fibroblasts (MEF) and in response to stimuli known to activate TBK1. Recombinant TBK1 is sufficient for multisite phosphorylation of purified Raptor *in vitro*, and genetic deletion, siRNA depletion, and chemical inhibition reveal that TBK1 is necessary for Raptor Ser877 phosphorylation in cells. While phosphorylation of Raptor Ser877 has been documented^16^, the function of this phosphorylation and the upstream signals that regulate this site remained poorly characterized. The data presented herein indicates that Raptor Ser877 phosphorylation limits mTORC1 signaling and this may represent a mechanism whereby TBK1 limits mTORC1 activity.

## Results and Discussion

### TBK1 phosphorylates Raptor in cell-free kinase assays

While the direct phosphorylation and activation of mTOR by TBK1 has been proposed^5^, other reports have described the negative regulation of mTORC1 by TBK1 without a clear mechanism^6^. Several reports have indicated that TBK1 physically interacts with multiple components of mTORC1^3,6^, indicating that TBK1 kinase activity might regulate a component of the mTORC1 complex in cells. We hypothesized that TBK1 suppresses mTORC1 by direct phosphorylation of a component of the mTORC1 complex.

**Figure 1:**
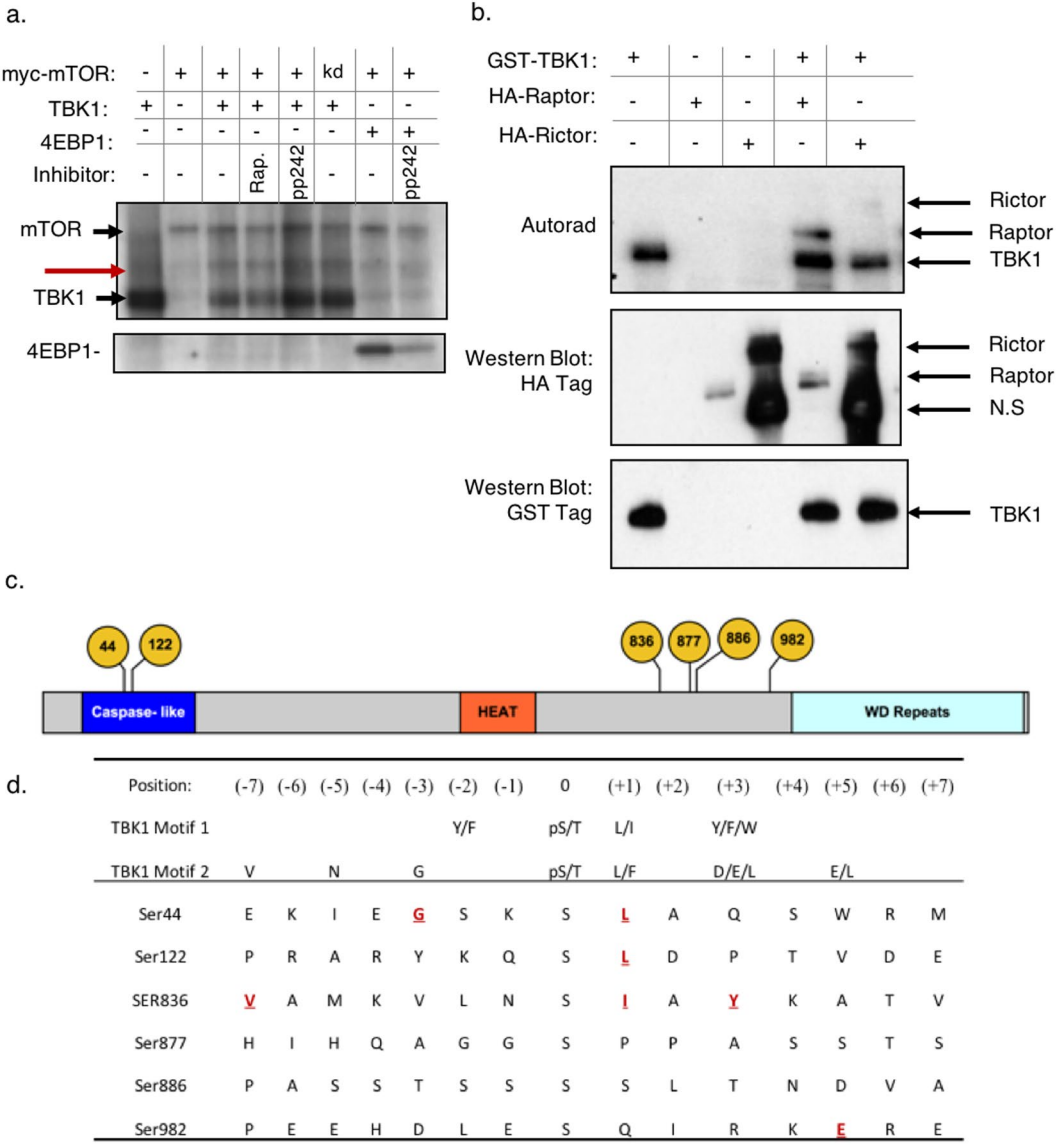
Identification of TBK1 dependent Raptor phosphorylation sites phosphorylation *in vitro*. (**a**) Myc-mTOR was immunoprecipitated from HEK293T cells under conditions that preserve mTORC1/2 complexes and subjected to i*n vitro* kinase assay containing ^32^P-γ-ATP with or without recombinant TBK1. mTORC1 activity was assessed by phosphorylation of 4EBP1. Indicated samples were pre-incubated for 30 minutes with the mTOR inhibitors rapamycin or pp242 (each 100 nM). Reactions were resolved with SDS-PAGE and autoradiograpy. The black arrows indicate which bands correspond to myc-mTOR and which correspond to TBK1. The red arrow indicates a putative TBK1 substrate. (**b**) As in A except that either HA-Raptor or HA-Rictor were immunoprecipitated and incubated with recombinant GST-TBK1. The black arrows indicate which bands correspond to which proteins (N.S = non-specific). (**c**) Domain structure of Raptor showing the positions of the phosphorylation sites identified by mass spectrometry. (**d**) Alignment of the primary amino sequence of the phosphorylation sites identified by mass spectrometry with the preferred TBK1 substrate consensus sequence. Residues that match the sequence are highlighted in yellow.

To test this hypothesis, we immunoprecipitated myc-mTOR from HEK293T cells under conditions that preserve mTORC1 and mTORC2 components and combined the resulting complex with recombinant TBK1 in the presence of radiolabeled ATP. The addition of TBK1 to the reaction did not increase the levels of ^32^P-γ-ATP incorporated into mTOR itself (Fig. 1a). However, recombinant TBK1 promoted phosphorylation of an approximately 150 kDa protein when incubated with mTOR immunoprecipitates (Fig. 1a, red arrow). This activity was not blocked by the addition of either of the mTOR inhibitors rapamycin or pp242, further suggesting that it was not due to mTOR kinase activity (Fig. 1a). Since mTOR was precipitated under conditions permissive for mTORC1 and mTORC2^17,18^ formation, we speculated that TBK1 phosphorylates an mTOR complex subunit in this size range. Two proteins, Raptor (149 kDa) and Rictor (192 kDa) were strong candidates as both proteins are known to be tightly and constitutively associated with mTOR. We immunoprecipitated tagged Raptor or Rictor from HEK293Ts and found that recombinant TBK1 showed activity towards Raptor but not towards Rictor (Fig. 1b).

Others have reported that TBK1 and IKKε can phosphorylate mTOR at Ser 2159 to promote its kinase activity^5^. That work screened a panel of recombinant kinases *in vitro* against an immobilized 32aa fragment of mTOR (aa2114-2175) fused to GST; Raptor was absent in this schema, and when TBK1 was tested against immunoprecipitated mTOR complexes, phosphorylation was measured with an antibody specific for phospho-Ser2159 mTOR. The presence of Raptor in our cell-free reactions may explain why we observed that recombinant TBK1 preferentially phosphorylates Raptor over mTOR in this context, as it may have served as a preferential substrate for TBK1.

To determine which sites on Raptor were phosphorylated in cell-free kinase assays, we performed a reaction as in Fig. 1a, except that unlabeled ATP was used in the reaction. Three reactions were performed: (1) HA-Raptor (2) HA-Raptor +ATP or (3) HA-Raptor +ATP and +TBK1. Each reaction was separated using SDS-PAGE, stained with Coomassie and the band corresponding to Raptor was excised, trypsin digested, enriched for phosphopeptides and then analyzed by liquid chromatography coupled to tandem mass spectrometry (LC-MS/MS). The peptides identified from the second reaction are presumed to be from another kinase that could be co-purified from cells with HA-Raptor, such as mTOR. In this way, we could tell which sites were phosphorylated specifically due to TBK1 activity and not a contaminating kinase that might co-purify with HA-Raptor. The phosphopeptides enriched in the third reaction were presumed to be due to TBK1 activity. In total, we identified five phosphopeptides that were enriched in the samples incubated with TBK1. The phosphorylation sites corresponded to Ser44, Ser122, Ser836, Ser877 and Ser982 (Table 1 and Fig. 1c). Three of the six phosphorylation sites had either leucine or isoleucine at the +1 position relative to the phosphorylation site, which matches the preferred substrate motif for TBK1^19,20^ (Fig. 1d). While TBK1 substrate motifs have been described, a significant portion of verified TBK1 substrates appear to lack this motif and are regulated by colocalization of substrate and kinase^1,19,21,22^. It may therefore be that the TBK1-dependent phosphorylation sites that match the motif are regulated by increases in TBK1 activity, whereas the others may be regulated by changes in TBK1 binding to Raptor.

**Table 1:** Phosphorylation Sites Identified Using Mass Spectrometry.

Peptide Sequence	Phospho Site (s)	m/z [Da]	Charge	ΔM [ppm]	Localization probability	Mascot score	Cell-Free normalized abundance			HEK293T normalized abundance		
							Control	(+ATP)	(+ATP + TBK1)	Control	PMA	PMA + AZ-5E
[39].IEGSKpSLAQSWR.[50]	S44*	721.346	2	0.93	100%	37	0.3	1.2	**1.5**			
[119].YKQpSLDPTVDEVK.[131]	S122*	801.377	2	0.57	100%	38	0.0	0.0	**1.0**			
[359].SYNCTPVpSpSPR.[369]	S366/S367*	674.274	2	0.05	50%	30	0.6	2.0	0.4	0.8	0.0	1.2
[691].NYALPpSPATTEGGSLTPVR.[709]	S696	1006.474	2	2.22	100%	56	1.7	1.0	0.3	0.0	**1.7**	0.3
[691].NYALPSPATTEGGSLpTPVR.[709]	T706	1005.981	2	1.32	100%	75	0.7	2.0	0.4	0.7	**2.0**	0.3
[719].SVSpSYGNIR.[727]	S722	531.735	2	0.6	100%	39	0.3	2.1	0.6	1.5	0.0	0.5
[833].VLNpSIAYK.[840]	S836*	494.249	2	0.06	100%	34	0.0	0.0	1.0			
[850].VLDTSSLTQpSAPApSPTNK.[867]	S859, S863	988.936	2	2.42	100%, 100%	88	0.9	1.3	0.9	1.1	**1.6**	0.3
[850].VLDTSSLTQSAPApSPTNK.[867]	S863	948.952	2	1.82	100%	104	0.9	1.6	0.5	1.1	**1.4**	0.5
[868].GVHIHQAGGpSPPASSTSSSSLTNDVAK.[894]	S877	891.415	3	0.66	100%	101	0.7	1.8	0.4	0.5	1.5	0.0
[868].GVHIHQAGGpSPPASSTSSpSSLTNDVAK.[894]	S877, S886	918.072	3	1.92	100%, 100%	77	0.0	0.5	2.4			
[974].IPEEHDLEpSQIR.[986]	S982	773.351	2	1.36	100%	42	0.0	0.0	**1.0**			
[1197].MALpSECR.[1203]	S1200*	473.679	2	0.07	100%	46	0.8	1.9	0.3			

Raptor phosphorylation sites detected in cell-free and HEK293T cells. Mass spectrometry (MS) data are derived from the highest scoring phosphopeptide across the samples. For confident phosphorylation site localization within a peptide, a probability (0–100%) is calculated based on the detected product ions as determined by the phosphoRS algorithm^41^. Mean-normalized quantitative data (peak areas) were calculated for each phosphopeptide in either cell-free or HEK293T experiments (values in bold indicate an increase upon TBK1 inclusion in cell free assays or depleted upon AZ-5E treatment in cells). An asterisk (*) denotes a phosphorylation sites not previously listed in the PhosphoSite database.

### TBK1 promotes Raptor Ser877 phosphorylation in cells and in cell-free kinase assays

**Figure 2:**
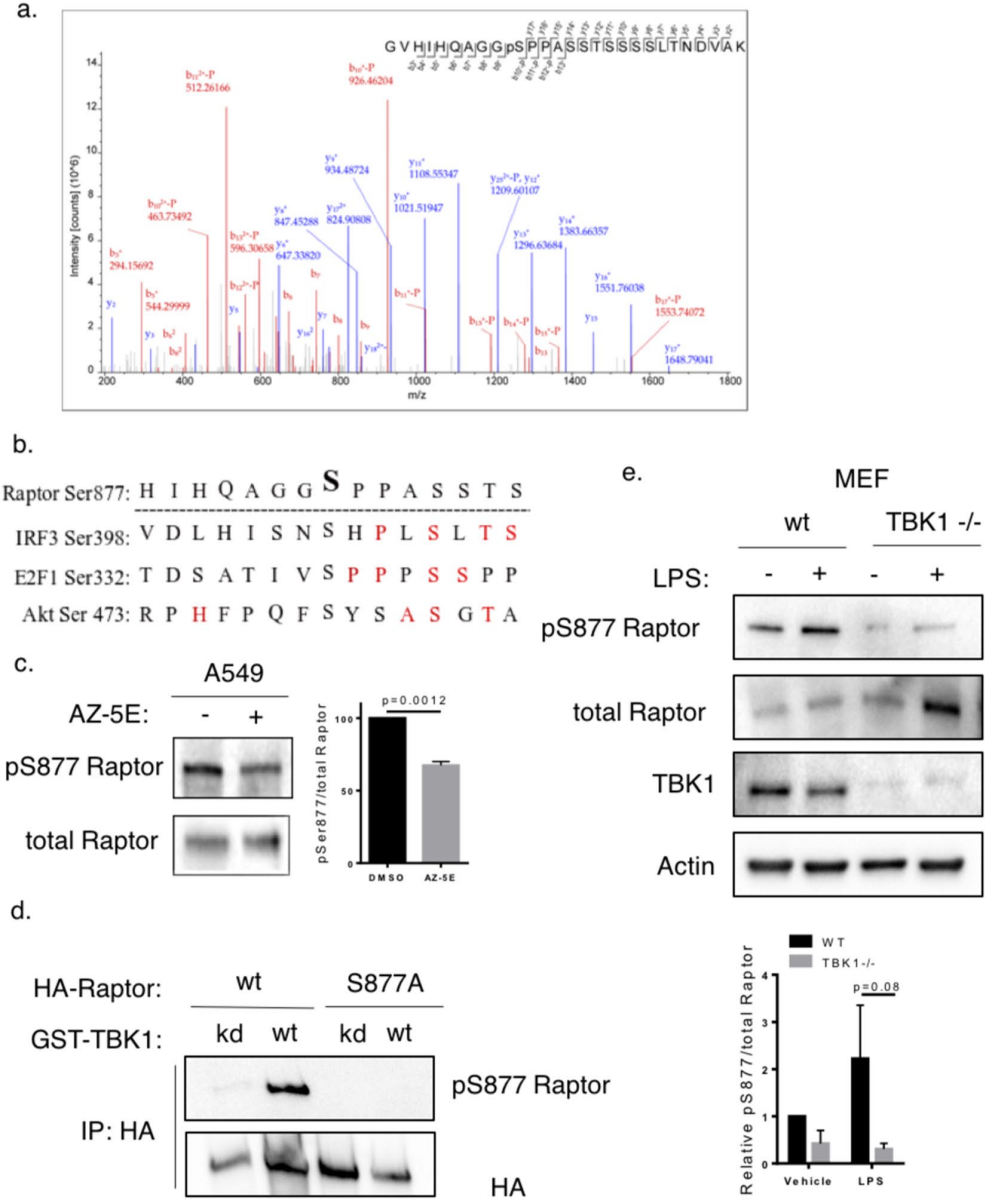
TBK1 promotes Raptor Ser877 phosphorylation in cells. (**a**) MS/MS spectra of the phosphopeptide containing phospho-Raptor Ser877 that was found to be less abundant in cells treated with the TBK1 inhibitor AZ-5E. (**b**) Alignment of Raptor Ser877 with known TBK1 target phosphorylation sites. The amino acids in red indicate amino acids that are conserved relative to optimized TBK1 motifs. (**c**) A549 cells were serum starved for 1 hour and then were treated with the AZ-5E (5μM) for 1 hour. Whole cell lysates were prepared and analyzed by western blotting with the indicated antibodies. Bar graph depicts ratio of phosphorylated:total Raptor, n = 4 independent replicates. (**d**) Either GST-wt or GST-kd TBK1 was overexpressed in HEK293T cells along with either HA-wt-Raptor or HA-S877A-Raptor. HA-Raptor was then immunoprecipitated and analyzed by western blot using either the phospho-Raptor Ser877 antibody or an antibody that recognizes the HA-tag. (**e**) Serum starved wild type or TBK1 knockout mouse embryonic fibroblasts were either untreated or treated with LPS for 1 hour. Whole cell lysates were prepared and then analyzed by western blotting with the indicated antibodies. Bar graph depicts ratio of phosphorylated:total Raptor, n = 3 independent replicates.

We next examined which of these candidate phosphorylation sites identified in cell-free kinase assays were dependent on TBK1 activity in intact cells. HA-Raptor was immunoprecipitated from HEK 293 T cells either unstimulated, stimulated with a known inducer of TBK1, phorbol myristoyl acetate (PMA)^23^, or a combination of PMA in conjunction with the TBK1 inhibitor AZ-5E^24^. As in Fig. 1, we excised the band that corresponded to Raptor and performed mass spectrometry. We identified five sites that were induced by PMA and blocked by AZ-5E (Table 1): Ser877, Ser863, Ser 859, Ser696, and T706, corresponding to both of the multisite phosphorylation clusters previously identified^14^. Only Ser877 was identified as TBK1 dependent in both cell-free kinase assays and in cells (Fig. 2a and Table 1). While we were unable to identify any of the other phosphorylation sites as TBK1 dependent in cell-free assays, these sites might be present in other cells or tissues or may be induced by different stimuli. Raptor Ser877 and the surrounding sequence share similarity with several known TBK1 target sites including IRF3 Ser398, E2F1 Ser332 and Akt Ser 473 (Fig. 2b), indicating that the amino acid sequence surrounding Raptor Ser877 is a strong candidate for TBK1 phosphorylation.

**Figure 3:**
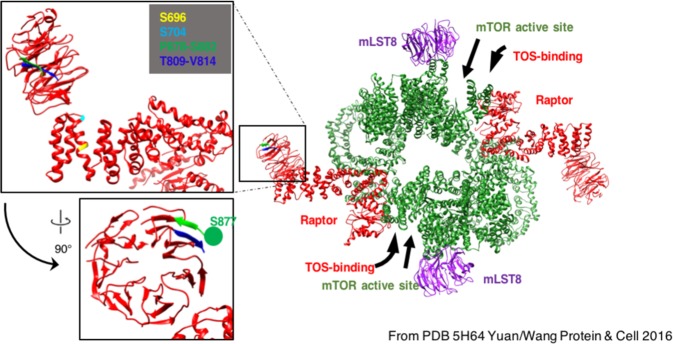
Location of TBK1 phosphorylation targets within the Raptor/mTORC1 structure. Cryo-EM structure derived from Yang *et al*.^34^ depicting mTORC1 complex with the position of TBK1-dependent Raptor phosphorylation sites. mTOR (green), mLST8 (purple), and Raptor (red) are depicted. The loop containing Raptor Ser877 is between the blue (T809-V814) and light green (P878-S882) strands of the distal WD40 domain. Raptor Ser696 and S704 on the adjacent armadillo domain are also depicted.

**Figure 4:**
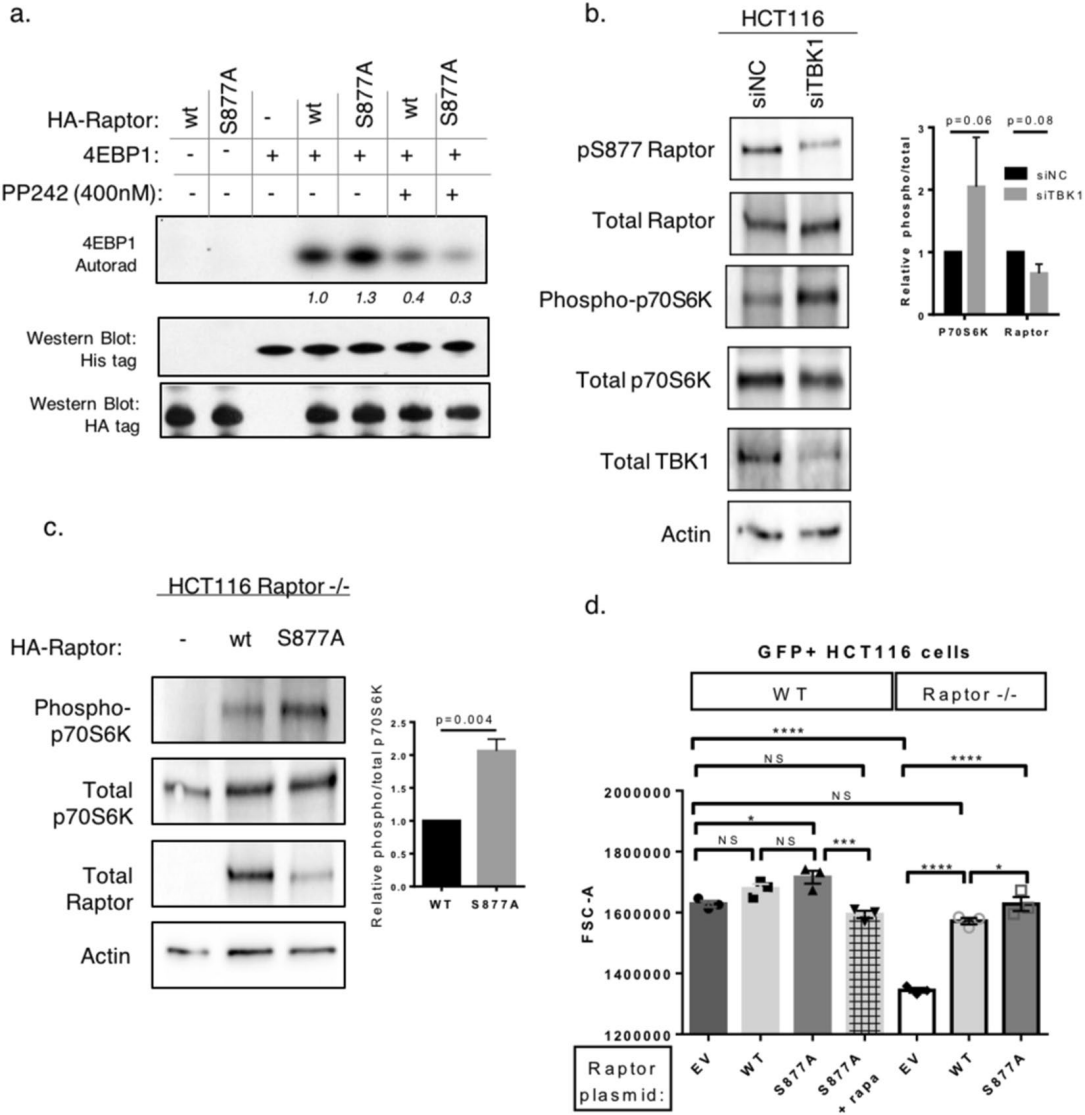
Expression of Raptor S877A is associated with greater levels of mTORC1 activity. Expression of Raptor S877A increases p70S6K phosphorylation. **(a)** HEK293T cells were transfected with either HA-tagged wt-Raptor or Raptor S877A. Raptor was then immunoprecipitated and a kinase assay was performed using His-tagged 4EBP1 as a substrate. This activity was blocked by the ATP competitive mTOR inhibitor PP242 as described in Fig. 1a. The italicized numbers beneath the autoradiograph indicate the relative level of phosphorylated 4EBP1 normalized to the total 4EBP1. (**b**) HCT116 cells were transfected with either siRNA either targeting a non-coding region (siN.C.) or TBK1 (siTBK1) for 48 hrs. Whole cell lysates were then prepared and analyzed with the indicated antibodies. Bar graph depicts ratios of phosphorylated:total P70S6k and phosphorylated: total Raptor, n = 3 independent replicates. (**c**) HCT116 Raptor −/− cells were transiently transfected with either empty vector (EV), wild type (wt) or S877A HA-Raptor. 48 hours after transfection whole cell lysates were prepared and analyzed by western blot. ratio of phosphorylated:total P70S6K, n = 3 independent replicates. (**d**) WT HCT116 or HCT116 Raptor knockout cells were transfected with plasmids encoding GFP and either empty vector control, wild type Raptor, or S877A Raptor for 72 hours. A subset of samples were treated for 48 hours with 100 nM rapamycin starting 24 hours after transfection. Cell size was determined by flow cytometry of forward scatter area (FSC-A) gated on live (low side scatter), GFP + singlet cells. 1000–20,000 GFP + cells per sample were analyzed. Bar graph depicts 3 independent experimental replicates and is representative of 4 independent experiments. Statistical significance was determined by ordinary one-way ANOVA with multiple comparisons with Tukey’s test. *p < 0.05; **p < 0.01; ***p < 0.001; ****p < 0.0001.

Of note, in cell-free kinase assays Raptor peptides with monophosphorylated Ser877 were detected in reactions that contained ATP but lacked recombinant TBK1 (Table 1). This may occur by several mechanisms. First, endogenous TBK1 may promote basal phosphorylation at this site prior to any stimulus, as we observe later in Fig. 2c,e. Second, given that several groups have found that Raptor can co-precipitate TBK1 in cells^3,6^, it is possible that endogenous TBK1 is present in the kinase reaction. Third, it is also possible that another kinase present in the reaction, such as mTOR, was responsible for the phosphorylation of Raptor Ser877. One study indicates that Raptor Ser877 phosphorylation was decreased in response to the mTOR inhibitors Torin 1 and rapamycin, and another demonstrated that Rheb overexpression could lead to increases in Raptor S877 phosphorylation, each consistent with the view that mTOR itself phosphorylates Ser877^25,26^. However, another study indicated that Raptor Ser877 phosphorylation was insensitive to Rapamycin or mTOR activity induced by insulin^14^. In the context of TBK1, we have found situations where inhibition of TBK1 led to an increase in mTOR activity but a decrease in the levels of Raptor Ser877 phosphorylation (see Figs 3a and 4b). This indicates that TBK1 does not increase Raptor Ser877 phosphorylation by increasing mTOR activity.

To validate the mass spectrometry findings, we used a phosphospecific antibody that specifically recognizes phosphorylated Raptor Ser877. In A549 cells, a 1 hour treatment with AZ-5E led to a decrease in Raptor Ser877 phosphorylation (Fig. 2c). Overexpression of wildtype (wt) TBK1 in HEK293T cells induced Raptor Ser877 phosphorylation (Fig. 2d), but overexpression of kinase-dead (kd) TBK1 K38A did not, suggesting that the kinase activity of TBK1 is required for Raptor Ser877 phosphorylation in this context (Fig. 2d). Notably, the signal from the antibody was highly specific to Raptor Ser877 phosphorylation as mutating Ser877 to Ala completely erased the signal (Fig. 2d).

In cell-free kinase assays, TBK1 strongly promoted the abundance of a doubly phosphorylated Ser877/Ser886 peptide, while in PMA-stimulated cells we observed primarily the AZ-5E sensitive accumulation of monophosphorylated Ser877.

Since TBK1 is known to be activated by pathogen-associated molecular patterns (PAMPS) such as LPS^1^, we hypothesized that treatment of cells with these agonists would induce Raptor Ser877 in a TBK1-dependent manner. We treated wildtype or TBK1 knockout MEFs (TBK1 −/−) with LPS for 1 hour after 1 hour of serum starvation. Under these conditions, TBK1 KO MEFs exhibited decreased basal and LPS-induced Raptor Ser877 phosphorylation as compared to wildtype (Fig. 2e). Interestingly, TBK1 −/− MEFs had greater levels of p70S6K Thr389 phosphorylation, both at baseline and in response to LPS. We focused on Thr389 p70S6K phosphorylation because it is a well-characterized and direct phosphorylation target of mTORC1, but it remains possible that TBK1 differentially regulates the various targets of mTORC1.

The finding that TBK1 limits mTORC1 activity induced by LPS contrasts with prior reports that TBK1 is required for mTORC1-mediated p70S6K Thr389 phosphorylation in response to LPS^5^. This difference may result from a difference in the serum starvation protocol used. Whereas the previous studies removed the serum overnight, we serum starved the cells for one hour prior to stimulation. Overnight serum starvation can induce many changes in cells grown in culture. For instance, serum starvation induces degradation of the Hippo pathway components YAP/TAZ^27^ and these proteins are known to modulate TBK1/IKKε-meditated activation of IRF3 in response to cytosolic DNA^28^. Moreover, YAP/TAZ can regulate mTOR activity by promoting the transcription of SLC38A1^29^. Another important consequence of serum starvation is cell-cycle arrest. Both TBK1 activity and Raptor Ser877 phosphorylation are known to be regulated by the cell cycle^30–32^, and overnight serum starvation induces cell cycle arrest.

We also observed that total Raptor protein levels were increased in TBK1 −/− MEFS that were stimulated with LPS (Fig. 2e). This suggests that TBK1, possibly through Raptor Ser877 phosphorylation, may negatively regulate Raptor protein abundance in certain contexts.

### Location of TBK1 phosphorylation targets within the Raptor/mTORC1 structure

To gain insight into the function of Raptor Ser877 phosphorylation, we located the position of Raptor Ser877 and other putative TBK1 targets within the larger architecture of the mTORC1 complex. Several structural studies have shown that Raptor adopts a Z-shaped conformation within mTORC1, with the Caspase-like Raptor N-terminal Conserved (RNC) domain packing against the mTOR kinase domain and promoting mTOR dimerization^33^. The RNC domain contains the TOS-binding domain that directs TOS-motif-containing substrates to the mTOR active site^33–36^. Moving towards the C-terminus of Raptor, an extended armadillo repeat domain extends away from the central mTOR dimer toroid and ends at a WD40 β-propeller. The structure of Yang *et al*.^34^ reveals that a disordered region (815–877) bounded by two β strands (809–814 and 878–882) defines the polyphosphorylated region containing Raptor Ser877 as well as other observed phosphorylation sites at Ser863 at Ser859 (Fig. 3). While Ser877 cannot be visualized directly, the adjacent residue (Pro878) is ordered in the structure and implies that Ser877 and the adjacent polyphosphorylated loop point away from the center of the WD40 propeller towards the armadillo domain. Likewise, Raptor Ser696 and Ser704, which we observed to be sensitive to the TBK1 inhibitor AZ-5E in cells (Table 1), are positioned on the surface of the armadillo domain pointing up towards the space bounded by the WD40 propeller and the edge of the mTOR dimer. Raptor Ser877, Ser696, and Ser704 are all located at the end of Raptor distal to the RNC, TOS-binding site, and mTOR active site, suggesting they are unlikely to contribute to substrate selectivity or mTOR:Raptor association (Fig. 3).

### Raptor Ser877 phosphorylation suppresses mTORC1 activity

To examine the function of Raptor Ser877 phosphorylation, mTORC1 complexes were immunoprecipitated using Raptor S877A or WT Raptor. mTORC1 complexes containing Raptor S877A had greater activity towards a well-established mTORC1 substrate, 4EBP1, in cell free kinase assays than wild type Raptor (Fig. 4a).

Since S877A Raptor was associated with greater levels of mTORC1 activity in cell-free assays, we hypothesized that S877 phosphorylation would limit mTORC1 activity in cells. Since the effect of TBK1 on mTORC1 signaling is apparently context-dependent, we first wished to identify growth conditions which TBK1 inhibited mTORC1^3^. HCT116 cells grown in full media transfected with siRNA targeting TBK1 led to an increase in the levels of phospho-p70S6K Thr389 and a decrease in the levels of Raptor Ser877 phosphorylation (Fig. 4b).

To determine whether Raptor Ser877 phosphorylation promotes or inhibits mTORC1 in HCT116 cells, we transiently expressed in CRISPR-edited Raptor knockout HCT116 cells either wildtype Raptor or mutant Raptor S877A. Expression of Raptor Ser877A promoted higher levels of p70S6K Thr389 phosphorylation relative to wildtype Raptor (Fig. 4b), confirming that Ser877 phosphorylation inhibits mTORC1 signaling.

Active mTORC1 is known to increase cell size via both activation of p70S6K and inhibition of 4EBP1^37^. We used cell size as marker of mTORC1 function to test the relative activity of WT and nonphosphorylatable S877A Raptor. Both wildtype and Raptor knockout HCT116 cells were transfected with either empty vector, WT Raptor, or Raptor S877A. All samples were cotransfected with GFP as a marker of transfection. When cell size was measured by flow cytometry, Raptor knockout HCT116 cells are clearly smaller than WT HCT 116 cells (Fig. 4d). WT Raptor increases cells size in both WT and Raptor null cells, though this effect is significant only in Raptor null cells, where WT Raptor effectively complements the knockout, returning those cells to the same size as WT cells transfected with EV plasmid (Fig. 4d). In Raptor null cells, Raptor S877A increases cells size to a greater extent than WT Raptor, signifying greater mTORC1 activity (Fig. 4d). Raptor S877A also increases the size of WT cells expressing endogenous Raptor, though this effect is less pronounced than in Raptor null cells. Finally, rapamycin treatment starting 24 hours after transfection completely blocks the effect of Raptor S877A (Fig. 4d), validating that the cell size effect of Raptor S877A is via mTOR activation (Fig. 4d).

Only one study has examined the phenotype of a Raptor Ser877 mutant^38^. In that study, expression of a phosphomimetic mutant of Raptor (Ser877D) did not affect the ability of Raptor to bind the Ragulator complex proteins. However, phosphomimetic S-E or S-D mutations only partly approximate serine phosphorylation, as they have only half of the negative charge of a true phosphoserine. It remains possible that phosphorylation of Raptor Ser877 disrupts binding with Ragulator or other proteins that regulate mTOR activity or substrate preference. Based on of the position of Raptor Ser877 within the mTORC1 structure, we hypothesize that this phosphorylation site is not involved in mTOR complex formation or in Raptor’s ability to promote mTOR substrate recognition. Phosphorylation of Ser877 may alter the conformation of Raptor, promote its interaction with other negative regulators of mTORC1 or alter the stability of Raptor protein to limit mTORC1 signaling.

**Figure 5:**
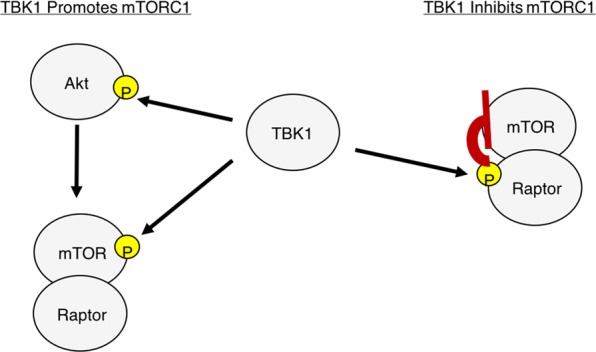
Model demonstrating the mechanisms of TBK1 mediated mTOR regulation.

Here, we present a novel mechanism whereby TBK1 can suppress mTORC1 signaling by promoting phosphorylation of Raptor Ser877. An important future direction will be to determine how TBK1 might switch between alternate modes of promoting Akt/mTOR activation and repressing mTORC1 activity by Raptor Ser877 phosphorylation (see model Fig. 5). One possibility is that TBK1 regulates mTORC1 in an alternating or sequentially ordered process in which TBK1 promotes mTOR activity by direct phosphorylation in response to a stimulus and at later time points promotes Raptor Ser877 phosphorylation as a self-limiting feedback mechanism. This will have implications for the implementation of TBK1 inhibitors in disease models. It will also be important to determine the biological relevance of TBK1-mediated mTORC1 repression by Ser877 phosphorylation in *in vivo* models.

## Materials and Methods

### Cell lines, plasmids, recombinant proteins

All cells were maintained in DMEM (4.5 g/L glucose) supplemented with 10% FBS and Penicillin/Streptomycin (Gibco). For serum starvation, cells were grown in serum-free media for 1 hour before the experiment. HEK293T and HCT116 cells were obtained from the UNC Tissue culture core facility. The wt and TBK −/− MEFs were as described previously^24^. pRK5-HA-Raptor and pRK5-myc-Rictor were obtained from Addgene (Plasmid #8513 and #1860). Genewiz performed the site directed mutagenesis of pRK5-HA-Raptor to generate an expression plasmid for Raptor S877A. The GST-Raptor 308–1019 was a kind gift from Dr. Pengda Liu (University of North Carolina at Chapel Hill). For immunoprecipitation experiments, HA-tag or Myc-tag antibody-conjugated agarose beads were purchased from Cell Signaling Technology. The phospho Raptor Ser877 antibody (09–107) was from Millipore, and all of the other antibodies were obtained from Cell Signaling Technology. The HCT116 CRISPR-edited Raptor knockout cells were a kind gift from Dr. Wenyi Wei (Beth Israel Deaconess Medical Center, Harvard Medical School). Recombinant TBK1 was purchased from Life/Invitrogen and SignalChem.

### Stimulation with immune modulators

MEFs were serum starved for 1 hour prior to stimulation with 10ug/mL of LPS. LPS was purchased from Invivogen (tlrl-b5lps).

### siRNA Knockdowns

siRNA targeting TBK1 was purchased from Dharmacon (Thermo Scientific). HCT116 cells were transfected using Dharmafect Reagent 1 (Thermo Scientific) according to the manufacturer’s protocol.

### Western blot analysis

Whole cell lysates were prepared by washing cells with phosphate buffered saline and then lysed in Lysis Buffer (40 mM HEPES pH 7.4, 2 mM EDTA, 10 mM pyrophosphate, 10 mM glycerolphosphate, 0.3% CHAPs, 1x complete EDTA-free protease inhibitor (Roche) and Phosphatase Inhibitor Cocktail 3 (Sigma Aldrich)) for 10 minutes on ice. The proteins were then separated using SDS-PAGE, transferred to nitrocellulose membranes and incubated with primary antibodies over night (see above). The signal from each primary antibody was measured the following day using horseradish peroxidase conjugated secondary antibodies (Promega) and visualized using Bio-Rad ChemiDoc western blot imager. The images obtained represent the maximum exposure time before the chemiluminescence signal was saturated. Where applicable, densitometry analysis was performed using ImageJ^39^.

### Kinase assay

HEK23T cells were transfected with Myc-mTOR, HA-Raptor or HA-Rictor using X-Treme gene transfection reagent (Roche). Two days after transfection, cells were lysed and incubated with the HA-antibody or Myc-antibody conjugated agarose beads for 1 hour. The beads were then washed three times with Lysis Buffer (without phosphatase inhibitors) and then in kinase assay buffer (1 mM beta-glycerolphosphate, 20 mM Tris pH 7.4, 12 mM MgCl_2_ and 100 μM ATP). The radiolabeled ATP was from Perkin Elmer and the unlabeled ATP was from Sigma Aldrich. The beads were then incubated with the TBK1, ATP or the appropriate negative control at 30 degrees for 30 minutes. The reaction was stopped using SDS-PAGE Laemmli buffer. His-tagged recombinant 4EBP1 was purchased from Sigma-Aldrich.

### Mass spectrometry

The mass spectrometry experiments were carried out at the UNC Proteomics Core Facility. Bands corresponding to Raptor were in-gel digested with trypsin overnight. Extracted peptides were enriched for phosphopeptides using TiO_2_. The TiO_2_ elution for each sample was analyzed by LC/MS/MS on a ThermoScientific Q-Exactive HF mass spectrometer. Samples were fractionated by C18 (Thermo PepMap RSLC) over a 45 min gradient from 5–35%B, where mobile phase A = 0.1% formic acid and mobile phase B = acetonitrile with 0.1% formic acid (ThermoScientific Easy nLC 1000). The top 15 most intense ions were chosen for HCD fragmentation. Data were searched against a reviewed Human UniProt database using Mascot. The parameters used were: 10 ppm precursor ion mass tolerance, 0.02 Da product ion mass tolerance, up to two missed trypsin cleavage sites, carbamidomethylation of Cys was set as a fixed modification and oxidation of M, deamidation of N, Q, and phospho of S, T, Y were set as variable modifications. A peptide false discovery rate of 5% was used to filter all results.

### Structural modeling

The structural model of the mTORC1 phosphorylation sites was constructed with UCSF Chimera using PDB coordinates 5H64^34,40^.

### Determination of cell size by flow cytometry

WT HCT116 and Raptor knockout HCT116 cells (1 × 105) were seeded in 24 well plates overnight prior to transfection with pEGFP (200 ng) and 500 ng of either empty vector (pCDNA3), WT Raptor, or Raptor S877A. After 24 hours, 100 nM rapamycin or vehicle was added to samples for 48 hours. At 72 hours, cells were washed, trypsinized, and collected for flow cytometry. Dead cells were gated away by high side scatter (SSC-A) and two singlet gates were used to exclude doublets and larger clumps. Transfected cells were gated by GFP signal and cell size was determined by sample median FSC-A. Identical results are seen with FSC-W. Data is indicative of 4 independent experiments.

## Data Availability

The proteomics data will be made available through the ProteomeXchange consortium using the PRoteomics IDEntifications (PRIDE) database.
